# Presence of *Akkermansiaceae* in gut microbiome and immunotherapy effectiveness in patients with advanced non-small cell lung cancer

**DOI:** 10.1186/s13568-022-01428-4

**Published:** 2022-07-06

**Authors:** Anna Grenda, Ewelina Iwan, Izabela Chmielewska, Paweł Krawczyk, Aleksandra Giza, Arkadiusz Bomba, Małgorzata Frąk, Anna Rolska, Michał Szczyrek, Robert Kieszko, Tomasz Kucharczyk, Bożena Jarosz, Dariusz Wasyl, Janusz Milanowski

**Affiliations:** 1 Department of Pneumonology, Oncology and Allergology, Medical University of Lublin, Jaczewskiego 8, 20-954 Lublin, Poland; 2 Department of Omics Analyses, National Veterinary Research Institute, Pulawy, Poland; 3 Chair and Department of Neurosurgery and Pediatric Neurosurgery, Medical University of Lublin, Lublin, Poland

**Keywords:** *Akkermansiaceae*, NSCLC, Response to immunotherapy, PD-1, PD-L1, Microbiome

## Abstract

The significance of *Akkermansia* bacteria presence in gut micobiome, mainly *Akkermansia mucinifila*, is currently being investigated in the context of supporting therapy and marker for response to immunotherapy in cancer patients. It is indicated that patients with non-small cell lung cancer (NSCLC) treated with immune checkpoint inhibitors (ICIs) respond better to treatment if this bacterium is present in the intestine.

We performed next-generation sequencing of the gut microbiome from patients treated in the first or second line therapy with anti-PD-1 (anti-programmed death 1) or anti-PD-L1 (anti-programmed death ligand 1) monoclonal antibodies. In our study group of 47 NSCLC patients, the percentage of *Akkermansiaceae* was higher in patients with disease stabilization and with partial response to immunotherapy compared to patients with disease progression. Moreover, we found that a higher percentage of *Akkermansiaceae* was present in patients with squamous cell carcinoma compared to adenocarcinoma. Our study showed that *Akkermansiaceae* could be supporting marker for response to immunotherapies in NSCLC patients, nonetheless further in-depth studies should be conducted in the role of *Akkermansiaceae* in cancer immunotherapy.

## Key points

- Composition of the microbiome can influence patients response to immunotherapy
- Response to immunotherapy of NSCLC patients is associated with the presence of *Akkermansiaceae* in the gut
- *Akkermansia* could be used as a predictor for patient treated with immunological checkpoint inhibitors

## Introduction

Immunotherapy with anti-PD-1 (Programmed Death 1), anti-PD-L1 (Programmed Death Ligand 1) monoclonal antibodies in patients with advanced non-small cell lung cancer (NSCLC) is the most modern method, prolonging patients life with limited side effects in both monotherapy and combination therapy (De Mello et al. 2021; Qu et al. 2021).

PD-L1 is a molecule that cancer cells use, by turning on its expression on their surface, to hide from the immune system (Chen et al. 2019). PD-L1 binds to PD-1 receptor on T cells and, in result, the immune system does not attack the cancer cells which allows cancer cells to avoid destruction (Chen et al. 2019; Cha et al. 2019). Immunotherapy with anti-PD-1 and anti-PD-L1 monoclonal antibodies blocks the PD-1/PD-L1 pathway, making tumor cells visible to the immune system and allowing it to activate immune factors leading to the elimination of cancer cells (Sun et al. 2020; Makuku et al 2021). The presence of the PD-L1 molecule expression on tumor cells determined by IHC (immunohistochemistry) is the only predictive factor for qualification to immunotherapy in NSCLC patients. However,it is not the only determinant of a response to immunotherapy. The effectiveness of immunotherapy also depends on the tumor mutations burden (TMB) or genetic predispositions such as the presence of a DNA repair deficiency (presence of microsatellite instability).

On the other hand, resistance to the treatment, despite of high expression of PD-L1 on tumor cells, is observed in some patients treated with immunotherapy and factors that could predict the resistance are being searched for (Sun et al. 2020; Topalian et al. 2019; Elkrief et al. 2019). Most patients with NSCLC develop primary resistance during immune checkpoint inhibitors (ICIs) monotherapy used in second line treatment and less than 20% of such patients achieve partial or complete response. However, ICIs used as first-line treatment in patients with PD-L1 expression on more than 50% of tumor cells cause the response in more than half of advanced NSCLC patients. Acquired resistance also occurs in initially responding, advanced NSCLC patients treated with immunotherapy (Topalian et al. 2019; Wang et al. 2020).

Increasing attention is being paid to the gut microbiome as a factor associated with the efficacy of ICIs treatment. Mention is made of the ‘driver microbiome’ for immunotherapy effectiveness. It has been found that *Ruminoccocaceae*, *Barnesiellaceae*, *Enterococcceae*, *Lactobacillae* and *Akkermansiaceae* bacteria may be of importance as drivers in immunotherapy (Liu et al. 2019; Zheng et al. 2019; Hakozaki et al. 2020). The last of the mentioned group is particularly interesting because researches show that presence of *Akermansiaceae*, mainly *Akermansia mucinifila*, in gut microbiome is a favourable factor for the response to immunotherapy in cancer patients (Routy et al. 2018, Zhang et al. 2020).

*A. muciniphila* is the most well-studied member of *Akkermansiaceae*. It is classified as probiotic and its reduced amount is associated with diseases such as obesity, diabetes, inflammation, and metabolic disorders. (Hansen et al. 2014; Roopchand et al. 2015; Earley et al. 2019). In colorectal cancer patients, it is described as one of the possible causes of this disease, due to its ability to degrade mucin in the intestine, causing inflammation and subsequent cancer (Weir et al. 2013; Sanapareddy et al. 2013). On the other hand, positive effect of its presence on the treatment of patients with colorectal cancer (CRC) is reported (Hou et al. 2021). Next generation sequencing (NGS) of 16S *rRNA* gene (V3-V4 regions) showed that the abundance of *Akkermansia muciniphila* is remarkably increased in the FOLFOX (fluorouracil and leucovorin with oxaliplatin) treated individuals and positively correlates with the therapeutic effect of chemotherapy (Zackular et al. 2013; Hou et al. 2021). In NSCLC and in renal cell carcinoma patients treated with anti-PD-1 antibodies, *A. muciniphila* seems to be enriched in responders compared to non-responders (Routy et al. 2018). Also, in hepatocellular carcinoma patients responding to anti-PD-1 treatment, the presence of *A. mucinifila* bacteria was observed (Zheng et al. 2019).

The *Akkermansia mucinifila* is called the next-generation beneficial microbe (Naito et al. 2018). It was found that *A. muciniphila* MucT directly involved in immune regulation and enhancement of trans-epithelial resistance by a highly abundant outer membrane pili-like protein (Ottman et al., 2017a, b). Proteins produced by *A. mucinifila* induced production of specific cytokines through activation of Toll-like receptor (TLR) 2 and TLR4 and it mainly leads to high levels of IL-10 (Ottman et al. 2017a). Moreover, occupation of the mucosa by *A. muciniphila* allows other beneficial microorganisms e.g. producing SCFA including butyric acid to colonize the intestinal wall (Ottman et al. 2017a, b).

In mouse tumor model, Routy et al. revealed that oral supplementation with *A. muciniphila* after fecal microbiome transplantation in mice not responding to therapy restored the efficacy of PD-1 blockade in an interleukin-12-dependent manner by recruitment of CCR9 + CXCR3 + CD4 + T lymphocytes into tumor beds. (Routy et al. 2018). In addition, it has been shown in mouse models that this bacterium may support the response to cisplatin-based chemotherapy in lung cancer patients, downregulating the levels of ki-67 (marker of proliferation), p53 (tumor protein 53), and FasL (Fas cell surface death receptor ligand) proteins and upregulating Fas (Fas Cell Surface Death Receptor) proteins, inducing the proinflammatory factors levels such as IFN-γ (Interferon-γ), IL-6 (Interleukin 6), and TNF-α (Tumor Necrosis Factor α) (Chen et al. 2020). These results suggests that the therapeutic efficacy of the combined treatment of *Akkermansia* and cisplatin is superior to the only cisplatin treatment, and would be a promising strategy for the treatment of lung cancer (Chen et al. 2020).

The aim of our study was to investigate the relationship between the presence of *Akkermansiaceae* in gut microbiome and the effectiveness of immunotherapy in locally advanced or advanced NSCLC patients received immunotherapy in the first or second line treatment.

## Material and methods

The material consisted of stool samples was collected from 47 consecutive patients with locally advanced or advanced non-small cell lung cancer qualified for first or second-line immunotherapy. Stool samples were collected and frozen at − 20 °C until genetic material isolation. Informed consent was obtained from all individual participants included in the study. All patients were tested for the presence of *EGFR* gene mutations and *ALK* gene rearrangements. PD-L1 protein expression on tumor cells was assessed by IHC method as a part of routine diagnostics. The studied group consisted of 30 (63.8%) men and 17 (36.2%) women. The median age was 66 years (min–max: 49–79 years, standard deviation [SD]: 7.06). Adenocarcinoma was diagnosed in 28 (60%) patients, and squamous cell carcinoma in 19 (40%) patients. The clinical and demographic data are presented in Table 1.

**Table 1:** Clinical and demographic characteristic of the patients included in the study

Characteristic		*Percentage of Akkermansiaceae*		*X*^*2*^ *p*-value
		Below median	Above median	
Age				
< 66 years (n, %)	21 (44.7)	15 (71)	6 (29)	**5.071**
≥ 66 years (n, %)	26 (55.3)	10 (38)	16 (62)	**0.02**
Gender				
Female (n, %)	17 (36)	10 (59)	7 (41)	*0.339*
Male (n, %)	30 (64)	15 (50)	15 (50)	*0.56*
Histopathology diagnosis				
AC (n, %)	28 (60)	19 (68)	9 (32)	**5.983**
SCC (n, %)	19 (40)	6 (32)	13 (68)	**0.014**
Stage				
IIIB (n, %)	8 (17)	6 (75)	2 (25)	*1.842*
IV (n, %)	39 (83)	19 (49)	20 (51)	*0.174*
PD-L1 (IHC)				
< 50% (n, %)	31 (66)	15 (48)	16 (52)	*0.844*
≥ 50% (n, %)	16 (34)	10 (63)	6 (37)	*0.358*
Line of treatment				
First line (n, %)	12 (25)	6 (50)	6 (50)	*0.066*
Second line (n, %)	35 (75)	19 (54)	16 (46)	*0.797*
Antibiotics before immunotherapy (applied up to 1–4 weeks prior to ICPs)				
No (n, %)	37 (79)	18 (49)	19 (51)	*1.441*
Yes (n, %)	10 (21)	7 (70)	3 (30)	*0.229*
Smoking				
No (n, %)	7 (15)	4 (57)	3 (43)	*0.052*
Yes (n, %)	40 (85)	21 (53)	19 (47)	*0.819*
Toxicity of immunotherapy				
No	33 (70)	18 (55)	15 (45)	*0.006*
Yes	14 (30)	7 (50)	7 (50)	*0.938*
Response to immunotherapy				
SD (n, %)	13 (28)	3 (23)	10 (77)	
PD (n, %)	16 (34)	12 (75)	4 (25)	**7.751**
PR (n, %)	18 (38)	9 (50)	9 (50)	**0.02**

Bolding have been used to indicate statistical significance

Italics were used to distinguish between columns with x-square and *p*-values

### DNA extraction

20 mg of stool sample was homogenized (FastPrep 24, MP Biomedicals) and treated with cocktail of lysozyme (10 µg/ml, A&A Biotechnology) and lysostaphin (2000 U, Sigma-Aldrich) for 30 min in 37 °C. Extraction was done by Maxwell RCS 48 (Promega) device according to RSC Tissue DNA Kit (Promega) protocol. Total DNA was measured using Qubit 3.0 (Thermo Fisher Scientific) with High Sensitivity DNA Assay (Thermo Fisher Scientific).

### Library preparation and next generation sequencing

Sequencing library was prepared with 15 ng of total DNA according to 16S metagenomics protocol (Illumina). Quality check of libraries was performed by gel electrophoresis (Fragment Analyzer with dsDNA 935 Reagent Kit, Agilent). Normalization was done by fluorimeter (Qubit 3.0 with High Sensitivity Assay; Thermo Fisher Scientific). Pair-end sequencing (2 × 300 bp with V3 kit, Illumina) was performed on MiSeq (Illumina).

### Sequencing data analysis

Quality check of data was done by FastQC. Composition of V3 and V4 regions of 16S *rRNA* gene was assets via Qiime 2.0 with Silva 138 database.

### Statistical analysis

Statistical analyses were performed using Statistica 13 (TIBCO Softwere, California, USA) and MedCalc (MedCalc Software Ltd, Ostend, Belgium) softwares. The Pearson chi square test (*χ*^2^) was used to assess the differences in the incidence of high (above the median) and low (belowe the median) percentage of *Akkermansiaceae* between different groups of patients. The Kruscal-Wallis (K-W) test was used to evaluate the differences in the percentage of *Akermansiaceae* in the groups of patients with different responses to immunotherapy. The U-Mann-Withney test (U-M-W) was used in post-hoc analysis to assess differences between groups. Kaplan–Meier analysis was used to analyze PFS (progression free survival) assessed from the moment of immunotherapy administration. A p-value below 0.05 was considered statistically significant.

## Results

### Abundance of Akkermansiaceae in different groups of NSCLC patients

A large variety in the percentage of *Akermansiaceae* among other bacterial families in gut microbiome was found in examined NSCLC patients. The median was 0.17% with a range from 0 to 57% (SD = 11.6).

**Fig. 1:**
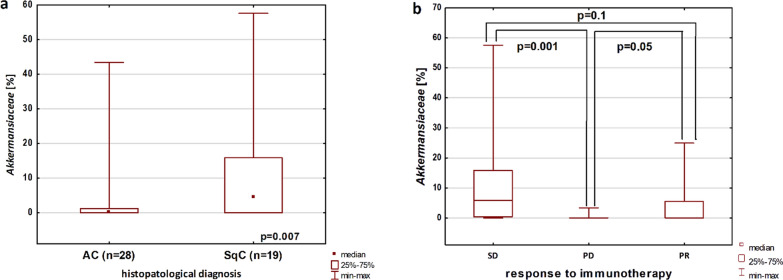
Differences in the percentage of *Akkermansiaceae* among other bacterial families determined by the NGS method: **a** in different histopathological subtypes of NSCLC, **b** in groups of patients with different responses to immunotherapy

In U-M-W test, we found higher abundance of *Akkermansiaceae* in squamous cell carcinoma (SCC) compared to adenocarcinoma (AC, p = 0.007, Fig. 1a). In group of patients who did not received antibiotics (up to 4 weeks prior immunotherapy, n = 37), we were sill observing higher percentage of *Akkermansiaceae* in SCC compared to AC patients (p = 0.02). In χ^2^ test, patients with SCC significantly more often had high abundance of *Akkermansiaceae* (percentage above the median) than patients with AC (χ^2^ = 5,983, p = 0.014). A high percentage of *Akkermansiaceae* (above the median) was also more frequently found in older patients (over 66 years of age) compared to younger patients (χ^2^ = 5.071, p = 0.02) (Table 1). Patients with and without prior antibiotic therapy (regardless of the pathomorphological diagnosis) had a similar abundance of *Akkermansiaceae*. Also, patients treated and not treated with antibiotics equally often showed high and low abundance of *Akkermansiaceae* in χ^2^ test (Table 1).

In K-W test, the analysis of percentage of *Akermansiaceae* in the groups of patients with stable disease (SD, n = 13), partial response (PR, n = 18) and disease progression (PD, n = 16) showed significant differences (H = 11.29, p = 0.003).

In post-hoc analysis (U-M-W test), a significantly higher abundance of *Akermansiaceae* in patients with SD compared to patients with PD was found (p = 0.001, Fig. 1b). Also, a higher percentage of this bacteria was observed in patients with PR than in patients with PD (p = 0.05, Fig. 1b). Patients who reached disease control (DC, PR + SD showed higher abundance of *Akermansiaceae* than patients with progressive disease (p = 0.003).

The same analyzes were performed after excluding 10 (21%) patients treated with antibiotics up to 4 weeks before immunotherapy. The differences were maintained with slightly less level of significance (SD vs. PD—p = 0.01, PD vs. PR—p = 0.07, DC vs PD—0.01). Moreover, χ^2^analysis showed that patients with SD or PR had significantly more frequently higher percentage of *Akkermansiaceae* (above the median) than patients with progressive disease (Table 1).

### Progression free survival depending on the abundance of Akkermansiaceae

Median PFS in whole group of patients was 14.3 months with 95% of Confidence Interval (CI) from 8.4 to 28.6.However, in 26 patients the data on the duration of PFS remained censored.

We did not observe any significant differences in PFS between groups in above and below the median percentage of *Akkermansiaceae* in gut microbiome. However, In patients with the first line of immunotherapy, PFS was insignificantly longer in the group with high percentage of this bacteria than in group of low abundance of *Akkermansiaceae* (8.3 months vs. 3.7 months, p = 0.2). Moreover, in squamous cell carcinoma, we observed insignificantly longer PFS in patients with percentage of *Akkermansiaceae* above the median compared to patients with abundance of this bacteria below the median (14.3 months vs 9.3 months, p = 0.1).

## Discussion

Currently, microbiome studies being conducted to tease out microbial predictors of treatment response in oncological patients. The composition of the gut microbiome in cancer patients including non-small cell lung cancer may be predictive factor of response to immunotherapy.

Examination of microbiome is not easy due to the requirement of NGS, which requires appropriate laboratory infrastructure and time-consuming and complex bioinformatics analysis. Nevertheless, ongoing research has shown that certain groups of bacteria play a crucial role in the treatment effectiveness in cancer patients including NSCLC. Among them is *Akkermansia mucinifila*, a major shareholder of the *Akkermansiaceae* group*.* It is likely that the microbiome differs in patients with different histopathological subtypes of lung cancer, with different molecular patterns, and with different metastatic mechanisms, followed by different therapeutic strategies (Huang et al. 2019). Huang et al. in study of the microbiome in patients with NSCLC strongly suggested differences in the microbiome composition in patients with different histopathologic types. However, they examined bronchoalveolar lavage and sputum, not feces.

Zheng et al. (Zheng et al. 2020) indicated that groups of bacteria observed in gut can differentiate the histopathological subtypes of NSCLC. Moreover, *Akkermansiaceae* were found in greater abundance in patients with metastatic disease compared to patients without metastases. The authors admitted that analyzed group were very small e.g. the SqC group consisted of only 3 patients. Nevertheless, it is important to report it, since very complex microbiome analysis involves many groups of bacteria, and any emerging differences should be verified by independent investigators, in a larger group of patients.

Zheng et al. studied NSCLC patients in early stages, whereas our study group included locally advanced or advanced NSCLC patients. We found that the *Akkermansiaceae* was more abundant in patients with squamous cell carcinoma compared to adenocarcinoma. Liu et al. found that *Akkermansia* was one of the dominant group (genera) in lung cancer patients with positive results of CYFRA-21-1 (Liu et al. 2019). CYFRA-21-1 is mainly expressed in epithelial-derived cells thus the expression in lung squamous cell carcinoma is higher than in lung adenocarcinoma (Fu et al. 2019). Despite the limitations of our study due to the small size of the research group, for the first time we showed direct differences in the presence of *Akkermansiaceae* in patients with different histologic types of NSCLC. However, our study should be further validated and confirmed by independent research with larger group of patients.

Routy et al. found that *Akkermansia* abundance positively correlated with partial response or stable disease during cancer immunotherapy. In mouse models, repletion of *Akkermansia* via oral gavage was able to repotentiate tumor response to anti-CTLA-4 and anti-PD-L1 therapy (Routy et al. 2018). Similarly. in our study we showed that abundance of *Akkermansiaceae* was higher in patients with disease stabilization or with partial response to immunotherapy compared to patients with disease progression. However, we did not show the effect of a high percentage of *Akkermansiaceae* on progression free survival. Although, we found a trend that high abundance of this bacteria promotes longer duration of PFS. The weakness of our study was not only the small size of the study group, but also the large proportion of patients whose observations were censored (26 of our patients were still in remission or we lost contact with them). This fact could have contributed to the inability to demonstrate differences in PFS and to the finding of a very high median of PFS in our study group (much higher than in clinical trials).

Interestingly, antibiotics use up to four weeks before immunotherapy did not significantly affects the obtained results. *Akkermansia mucinifila* is described as an antibiotic resistant bacteria (Dubourg et al. 2013). Resistance to antibiotics is one of the features of probiotics, and for this reason, it is classified as the next-generation probiotic, which could influence response to immunotherapy.

Lung cancer patients treated in first line with chemotherapy with a higher relative abundance of *Akkermansia* at baseline were less likely to have a gastrointestinal reaction caused by chemotherapy. Moreover, patients with relatively high abundance of *Akkermansia* were less likely to experience disease progression (Zhang et al. 2020). There are reports that presence of *A. mucinifila* in gut microbiome could prolongs PSF or OS in patients who receive immunotherapy (Derosa et al. 2018, 2021; Routy et al. 2018). *Derosa* et al. showed that high parentage of *A. mucinifila* among other bacteria in microbiome was associated with favorable clinical outcomes in immunotherapy treated patients, regardless of antibiotic use. The authors showed that the percentage of NSCLC or renal cell cancer patients without progression for 3 and 6 months was higher in group with high abundance of *A. mucinifila* (Derosa et al. 2018). The recent study of Derosa et al. put *A. mucinifila* in a somewhat new light. They found that the variability in the amount of this bacterium in the group of NSCLC patients varied greatly (from 0.0022% to 64.78%,) with a tendency towards high abundance of *A. mucinifila*. 75% of the examined population (seventy-fifth percentile) had 4.42% or more of *A. mucinifila* in the studied microbiome. This is consistent with our study, however it should be considered, that our method was not sensitive enough to confirm the presence of *A. mucinifila* species specifically, but to reliably detect presence of the *Akermansiaceae* genus. Derosa et al. showed that relative abundance of *A. mucinifila* ranging from > 0% to < 4.42% in the stool at diagnosis was associated with prolonged survival regardless of PD-L1 expression and performance status according to ECOG scale (Eastern Cooperative Oncology Group). This subgroup was associated with more inflammatory tumors with infiltration of T lymphocyte with upregulation of CD3ε (T-Cell Surface Glycoprotein CD3 Epsilon Chain), interferon gamma, and VCAM-1 (Vascular Cell Adhesion Protein 1). Conversely, patients with an *Akkermansia mucinifila* overrepresentation > 4.42% experienced higher rate of PD and shorter OS (Derosa et al. 2021). Researchers indicated that stratification based on the relative abundance of *Akkermansia* represents a more accurate independent predictor than the binary modality.

Further in-depth studies should be conducted to confirm the role of *Akkermansiaceae* in immunotherapy efficacy in order to develop of optimal strategy treatment in cancer. The presence of *Akkermansiaceae* in gut microbiome seems to be a favorable predictive factor for the effectiveness of immunotherapy in NSCLC patients. However, too high abundance of this bacteria in the microbiome may not be as beneficial. Very high percentage of *Akkermansiaceae* may be an indicator of dysbiosis, e.g. after antibiotic therapy. This may be related to the lack of microbiome variety which is most important for stimulating the immune system in cancer. Therefore, new studies are needed to determine the most favorable percentage of *Akkermansiaceae* in the gut microbiome. Continuation of research into the diversity of the microbiome (number of different strains of bacteria in the intestine) as a predictive factor for cancer immunotherapy are also necessary.

## Data Availability

The datasets generated and analysed during the current study are available from the corresponding author on reasonable request.
